# The effect of steam sterilization on different 3D printable materials for surgical use in veterinary medicine

**DOI:** 10.1186/s12917-021-03065-8

**Published:** 2021-12-23

**Authors:** Philipp Dautzenberg, Holger A. Volk, Nikolaus Huels, Lena Cieciora, Katharina Dohmen, Matthias Lüpke, Herman Seifert, Oliver Harms

**Affiliations:** 1 Department of Small Animal Medicine and Surgery, University of Veterinary Medicine Hannover, Bünteweg 9, 30559 Hannover, Germany; 2 Department of General Radiology and Medical Physics, University of Veterinary Medicine Hanover, Bischofsholer Damm 15, 30173 Hannover, Germany

## Abstract

**Background:**

Different 3D-printed materials polyactic acid (PLA), polyamide (PA), polycarbonates (PC), acrylonitrile butadiene styrene (ABS) and GreenTEC Pro®^I^ have been considered for surgical templates, but there is a sparity of data about how these materials are affected by steam sterilization. The aim of the current study was to test if and how these materials change morphologically when high temperature, pressure and humidity are applied during the steam sterilization process. The overall aim is to create patient-specific sawing templates for performing corrective osteotomies. After the designing process, test-specimens with five different materials: PLA, PC, ABS, PA and GreenTEC Pro® were 3D-printed in two filling grades (30 and 100%). The FDM method was used for printing. After 3D-printing, the test-specimens were steam sterilized with a standard program lasting 20 min, at a temperature of 121 °C and a pressure of 2–3 bar. In order to measure the deviation of the printed model, we measured the individual test-specimens before and after steam sterilization using a sliding gauge.

**Results:**

PC, PA and ABS showed great morphological deviations from the template after 3D-printing and steam sterilization (> 1%) respectively. ABS proved unsuitable for steam sterilization. PLA and GreenTEC Pro® demonstrated fewer morphological deviations both before and after sterilization. Therefore, we decided to perform a second test just with PLA and Green-TEC Pro® to find out which material has the highest stability and is probably able to be used for clinical application. The smallest deviations were found with the GreenTEC Pro® solid body. After autoclaving, the specimens showed a deviation from the planned body and remained below the 1% limit.

**Conclusion:**

Steam sterilization causes morphological deviations in 3D printed objects. GreenTEC Pro® seems to be a suitable material for clinical use, not only for intraoperative use, but also for precise modeling. Microbiological examination, as well as biomechanical tests, should be performed to further assess whether intraoperative use is possible.

**Supplementary Information:**

The online version contains supplementary material available at 10.1186/s12917-021-03065-8.

## Background

3D-printing is a rising technology in the field of personalized medicine becoming more readily available and affordable. In veterinary medicine, 3D-printing is also becoming more popular and gaining in importance [1]. Different printing techniques are described, the most common ones being Stereolitography (SLA), Fused Deposition Modeling (FDM), Selective Laser Sintering (SLS), Laminated Object Manufacturing (LOM) and PolyJet Technology [2]. To date, 3D-printing has been used in veterinary medicine in different fields, e.g. anatomical and surgical teaching, in veterinary orthopaedics, neurosurgery, oral and maxillofacial surgery [1]. Many techniques have been copied from human surgery.

3D printed templates or implants have been increasingly used in veterinary medicine. The material used varied and only small case numbers have been reported [3–11]. However, despite the promising reported surgical accuracy, very little is known how the material changes during printing and the steam sterilization process. Printability, mechanical properties, e.g., autoclavibility and robustness, biocompatibility and economic aspects are crucial when using 3D printing routinely in veterinary medicine [2].

Different studies in human medicine recommend to only use FDA-approved solution for 3D printing [12, 13]. Shaheen et al. have tested the effect of steam sterilization and gas plasma sterilization on 3D printed materials in a pilot study and postulate that most FDM printed materials show greater morphological variations in steam-pressure sterilization and that gas plasma sterilization is the more suitable option [14]. Furthermore, they mention in their study that the sterilization of plastics is still relatively unexplored, although the field of 3D- printed metals has already been extensively studied in science.

In another study, steam sterilization had no effect on the 3D printed material [15]. Twenty-seven surgical templates were measured with an intraoral scanner before and after steam-heat sterilization. The surgical guides were produced with the help of an SLA printer and from the material Dental SG Resin. Both studies differed in the number of objects to be examined. Marei et al. claim that their study gives a better significance due to the larger number of objects to be examined. Török et al. also found no significant morphological changes after steam sterilisation. They investigated a polyjet photopolymer (Objet MED610) for its sterilisability and used different sterilisation methods for this purpose [16]. A summary of the studies mentioned can be found in Table 1.

**Table 1:** Summary of the already mentioned previously published studies: material, printing process and steam sterilization

studies	material	3D-printing-method	steam sterilization
Marei et al. [15] (MAREI et al. 2019)	Dental SG	SLA	possible
Török et al. [16] (TÖRÖK et al. 2020)	MED610	Poly Jet	possible
Shaheen et al. [14] (SHAHEEN et al. 2018)	not stated	Poly Jet	not possible

In the current study five different 3D printable materials polyactic acid (PLA), polyamide (PA, nylon), polycarbonates (PC), acrylonitrile butadiene styrene (ABS) and GreenTEC Pro® were tested for heat resistance to a standard steam sterilization technique to evaluate whether these materials could be used clinically as surgical templates. The results of the study could provide a basis for the safe future use of 3D-printed templates in the various clinical settings.

## Results – morphological assessment

### Pre-test

Our pre-test showed a lack of morphological properties of PC, PA and ABS in both filling levels. Moderate surface deformations were found on PC, which were even more pronounced on ABS. The bodies have warped and could no longer be measured with a slide-gauge. After the autoclaving process, PA showed a change in the state of aggregation, the surface being slightly greasy and thus unsuitable for prompt intraoperative use.

If the test specimens already showed gross optical distortions, they were removed from the study for further tests.

Only PLA and GreenTEC Pro® showed dimensional stability suitable in our pre-tests for further investigations.

### Results of the dimensional stability tests of the materials

#### Before autoclaving

In the second run, five test specimens each in the filling grades 30 and 100% of the materials PLA and GreenTEC Pro® were printed (*n* = 20). The PLA with 30% filling showed the following results. After printing, the median deviation from the initial length was + 0.26%, from the initial width +  0.65%, from the initial depth +  3.1% and from the initial edge length - 1.1%. For the PLA test specimens with a 30% filling, the median values of the length were 50.13 mm, the width 40.27 mm, the depth 10.31 mm and the edge length of the integrated square 9.89 mm. After printing, PLA with a 100%-filling showed a median percentage deviation from the initial length +  0.84%, from the initial width +  1.63%, from the initial depth +  8.1% and from the initial edge length - 4.7%. For the PLA test specimens with a 100% filling, the median values of the length were 50.42 mm, the width 40.62 mm, the depth 10.80 mm and the edge length of the integrated square 9.51 mm.

**Fig. 1:**
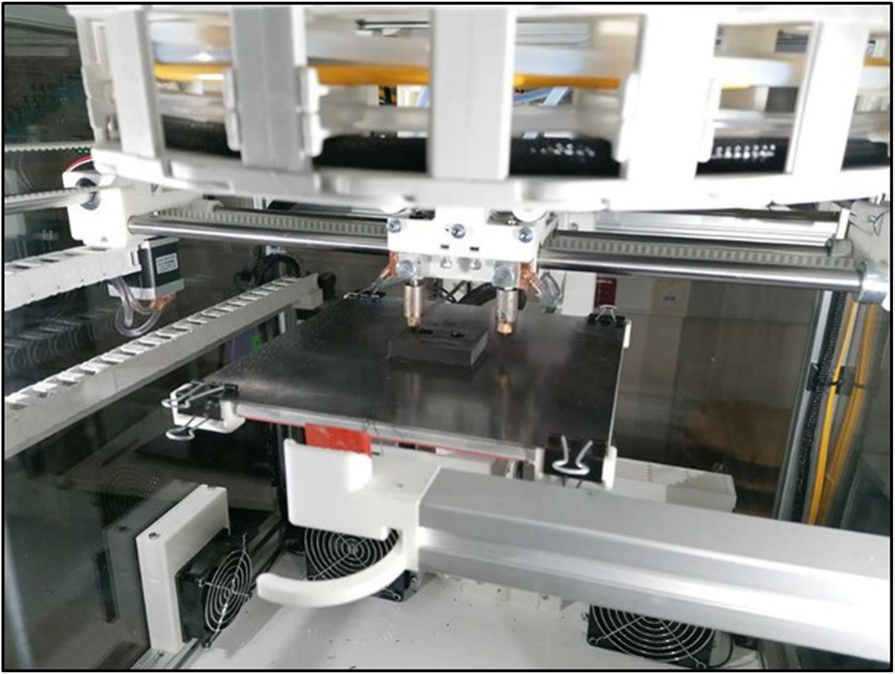
Results in percent of morphological deviations of 3D printed test objects after printing process

GreenTEC Pro® in the 30% fill showed a median deviation from the initial length - 0.18%,

+ 0.2% of the initial width, + 1.6% of the initial depth and +  0.1% of the initial edge length after printing. For the GreenTEC Pro® with a 30% filling, the median values were 49.85 mm in length, 40.05 mm in width, 10.17 mm in depth and 10.03 mm in the edge length of the integrated square. After printing, the GreenTEC Pro® solid showed a deviation from the initial length of - 0.12%, from the initial width +  0.3%, from the initial depth +  0.9% and from the initial edge length of the integrated square - 0.6%. For the GreenTEC Pro® with 100% filling, the median values were 49.98 mm in length, 40.08 mm in width, 10.08 mm in depth and 9.96 mm in the edge length of the integrated square (Fig. 1).

#### After autoclaving

The length of the test specimen made of PLA material with a 30% filling was reduced by - 3.19% on average, the width by - 3.15% on average, the depth by - 0.58% on average and the edge length by - 0.30% on average. After autoclaving the PLA test specimens with a 30% filling, the median values of the length were 48.45 mm, the width 38.98 mm, the depth 10.20 mm and the edge length of the integrated square 9.88 mm.

The length of the test specimen made of PLA material with a 100% filling was reduced by - 0.52% on average, the width by - 0.42% on average, the depth increased by + 0.83% on average and the edge length was reduced by - 0.84% on average. The body appeared to have become smaller in its entirety. After autoclaving, the PLA test specimens with a 100% filling showed median values in the length of 50.13 mm, in the width of 40.48 mm, in the depth of 10.89 mm and in the edge length of the integrated square of 9.44 mm.

After autoclaving, GreenTEC Pro® in the 30% filling showed an average deviation of - 0.22% in length, − 0.12% in width, + 0.39% in depth and - 1.1% in the edge length of the square compared to the planned object. After autoclaving, the GreenTEC Pro® material in the 30% filling showed median values in the length of 49.74 mm, in the width of 39.99 mm, in the depth of 10.21 mm and in the edge length of the integrated square of 9.96 mm. The test specimen with a 100% filling showed after autoclaving, the average deviation in length was - 0.1%, in width - 0.1%, in depth - 0.1% and in the edge length of the square - 0.6%.

**Fig. 2:**
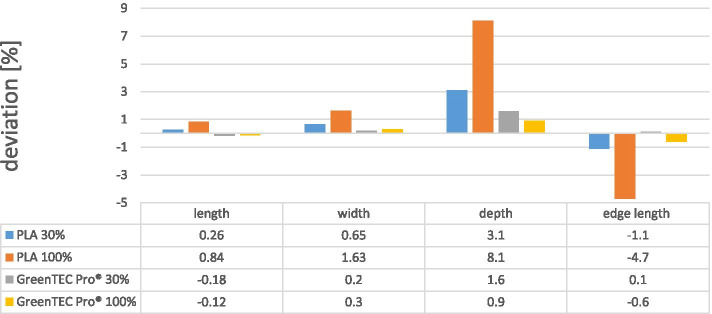
Results in percent of morphological deviations of 3D printed test objects after sterilization

After autoclaving, the specimens made of GreenTEC Pro® showed median values of 49.93 mm in length, 40.06 mm in width, 10.08 mm in depth and 9.91 mm in the edge length of the integrated square (Fig. 2).

The descriptive statistics can be seen in supplementary Table 3.

## Discussion

In veterinary medicine, 3D printed implants are becoming increasingly important and affordable. Dogs have large breed specific variations in size and shape and 3D printing provides the ideal solution to adapt an implant to the individual patient. Due to the nature of 3D printed plastic materials, autoclaving is often an obstacle because the printed forms can quickly change morphologically. Finding a material that is biocompatible, robust, environmentally friendly and inexpensive poses even greater challenges for 3D printing. None of the 3D printed material used in this study deviated less than 1% to the computer planned template, and should therefore not be seen as clinical useful according to Cone et al. [17]. The only material which deviated less than 1% in size after steam sterilization was GreenTEC Pro®.

GreenTEC Pro® with a filling of 100% seems to be a suitable material for precise 3D modeling and because of its robustness after the autoclaving process may be appropriate for intraoperative use. GreenTEC Pro® is a high-performance biopolymer made from renewable raw materials and might therefore be the only material to be further considered for clinical use. The material is inexpensive, completely biodegradable and compostable. It is a medium-hard, but at the same time slightly flexible material that can withstand loads and possesses very good mechanical and thermal loading properties. Furthermore, the material is food safe and odourless [18].

GreenTEC Pro® fulfills the requirements of autoclavability, biocompatibility and economic aspects for clinical use. Robustness must be tested in a cadaver study to find out if an intraoperative use, e.g., as saw guide for corrective osteotomy surgery is possible.

The choice and number of different materials limits the study and further studies with larger case numbers should be investigated. The right print settings for the material are essential for a precise print result. These settings may differ in some cases from the manufacturer’s specifications. Sometimes several test prints are necessary to achieve an accurate template. We tried various settings, but could still not get less than 1% deviations. Furthermore, different filling levels should also be tested in order to weigh up between robustness and material costs, as well as weight reduction.

In order to objectively assess the possible morphological deviations with high detail resolution, other technical devices, e.g., laser-technology or a micro CT examination before and after autoclaving are useful. Even small changes to the internal structure of the test body can be described and a computer-assisted measurement is feasible using this imaging technique.

## Conclusion

3D printing is gaining in importance in veterinary surgery, giving surgeons greater possibilities for an individualised patient solution. GreenTEC Pro® with a filling of 100% seems to be a suitable material for precise 3D modeling and because of its steam sterilization resistance may be appropriate for surgical use. Furthermore, it fulfills economic and ecological aspects that could facilitate its clinical use. Steam sterilization needs to be considered when developing new 3D printed surgical implants. Whether the material can be used as a surgical template for corrective osteotomies requires further microbiological and biomechanical investigations.

## Materials and methods

### Design of the test bodies

With the help of the software FreeCAD 0.18^II^, we designed a rectangular test body with an integrated square, which were used for measurements. The total edge lengths of the body were 50 mm × 40 mm × 10 mm with an edge length of the square being 10 mm × 10 mm.

### Materials

In our study, we tested five different 3D printable materials: PLA, PA, PC, ABS and GreenTEC Pro®. Each material was printed five times in two filling grades (30 and 100%).

Two test runs were performed. For the preliminary tests, two test specimens of each material were first printed. The first test specimen was made with a 30% honeycomb-like filling, i.e. the space between the outer walls of the specimen was filled with thin walls forming a hexagonal pattern. The proportion of plastic in the interior was 30%, while the remaining 70% was filled with air. The second test specimen was printed with a 100% plastic filling as a solid specimen. The test specimens in which the material already showed macroscopically recognisable deformations or changes in aggregate state after autoclaving were excluded for further investigations. The test bodies made of a material that appeared macroscopically stable after autoclaving were then printed again. Ten test specimens of each material were produced again, five of them with a 30% filling and five as solid specimens. Table 2 shows the material properties of GreenTEC Pro®.

**Table 2:** Material Data Sheet GreenTEC Pro® [17]

***Test***	***Method***	***Value/Unit***
Flexural modulus	ISO 527	4400 MPa
Tensile strength	ISO 527	61 MPa
Elongation at break	ISO 527	3,4%
Elongation at break	ISO 527	50 MPa
Elongation at impact	ISO 527	3%
Notched impact strength	ISO 179-1/1 eA	4,4 kj/m^2^
Impact resistance	ISO 179-171 eU	72kj/m^2^
Melt flow index (MFR)	ISO 1133	12 g/10 min
Melting temperature	ISO 3146-C	190–210 °C
VICAT A (VST)	ISO 306	160 °C
Dimensional stability (HDT/B)	ISO 75	115 °C
Shrinkage	ISO 294-4	0,4%
Tightness	ISO 1183	1,39 g/cm^3^

### 3D printing process – FDM printing method

The FDM method was used in the study. The principle of FDM is relatively simple. Filament plastic is heated and applied in layers to be processed on a heated plate. The process is protected by a chamber and cooled down so that the plastic can stiffen again [19]. Especially the setting of the first layer is important because this significantly influences the stability of the printed object. The first layer determines how successful the printing is.

**Fig. 3:**
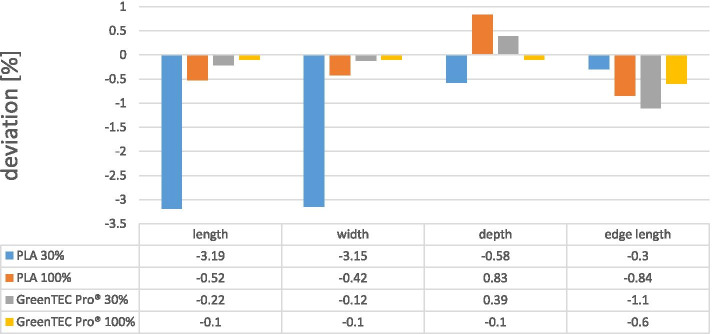
FDM printing process of a test specimen made of the GreenTEC Pro® material

In our study, we used the HT500 FDM printer^III^ (Fig. 3).

### Steam sterilization

Steam sterilization takes place in a pressure-resistant chamber, the so-called autoclave. Autoclaving can be divided into four steps. The first section is the climb time. During this time the interior of the autoclave is vented. During this process the atmospheric air is displaced from the interior and replaced by saturated, strained water vapour. The venting is carried out in a flow process or by fractional venting, after complete venting the venting valve is closed. After this time, the material to be sterilized also reaches the required temperature at any point by the action of saturated steam. Then the actual sterilization phase begins. The duration of the sterilization depends on the germ load and the sterilization temperature. After the sterilisation time, the cooling phase begins and thus the end of the autoclaving cycle. The reduction of postoperative complications is achieved by steam heat sterilization, as the microbial load is reduced, thus lowering the risk of infection [20, 21]. Using a Selectomat PL1^IV^, for plastics, a standard program lasting 20 min is run at the Clinic for Small Animals, University of Veterinary Medicine Hannover, Hannover, Germany, at a temperature of 121 °C and a pressure of 2–3 bar.

### Measurement

To assess the accuracy of 3D printing and the subsequent autoclaving process, three investigators measured independently on three different days all twenty test specimens, including five 30% filling PLA and GreenTEC Pro® and five 100% filling PLA and GreenTEC Pro® using a sliding gauge (n per tester and material = 45).

The measurements of the printed test specimens were compared with the data of the planned test specimen and the autoclaved test specimens with the printed specimens respectively. The percentage difference was determined and evaluated.

According to the publication of Cone et al., divergences above 1% are to be considered excessive and therefore unsuitable for clinical use [17].

### Statistical methods

The statistical analysis was performed using the SAS Enterprise Guide 7.1 software. The measurement data of the printed test specimens were compared with the data of the planned test specimen and the autoclaved test specimens with the data of the printed test specimens. The percentage difference between the values was determined and statistically evaluated.

## Companies mentioned

I) *Copyright© 2019 Extrudr | FD3D GmbH*
II) *Jürgen Riegel, Werner Mayer, Yorik van Havre, FreeCAD, Version 18.0, 2019*
III) *Kühling&Kühling GmbH, Christianspries 30, D-24159 Kiel, Germany*
IV) *MMM Group; Selectomat PL, Programm Kunststoff, 01004494571130; Serial-No.: EB090220, Semmelweisstraße 6, D-82152 Planegg/München, Germany*
V) *Connex®, Conmetall Meister GmbH, Hafenstraße 26, D-29223 Celle, Germany*

## Supplementary Information

**Additional file 1: Table 3.** Representation of the median values and mean percentage deviations from the planned standard object. Representation of the materials PLA and GreenTEC Pro®, in the filling degrees 30 and 100% (*N* = total number of measurements).

## Data Availability

The datasets used and/or analysed during the current study are available from the corresponding author on reasonable request.

## Abbreviations

3D: Three-dimensional
ABS: Acrylonitrile butadiene styrene
FDA: US Food and Drug Administration
FDM: Fused Deposition Modeling
PA: Polyamide
PC: Polycarbonates
