# Supplementary material for: The effect of steam sterilization on different 3D printable materials for surgical use in veterinary medicine
Source: BMC Vet Res. 2021 Dec 23;17:389. doi: 10.1186/s12917-021-03065-8 (PMC8697434; doi:10.1186/s12917-021-03065-8)
Supplement: Supplementary file 1 — Additional file 1: Table 3. Representation of the median values and mean percentage deviations from the planned standard object. Representation of the materials PLA and GreenTEC Pro®, in the filling degrees 30 and 100% (N = total number of measurements). [file 12917_2021_3065_MOESM1_ESM.docx]

Table 3. Representation of the median values and mean percentage deviations from the planned standard object. Representation of the materials PLA and GreenTEC Pro®, in the filling degrees 30% and 100% (N = total number of measurements).

| ***ID*** | **N** | **variable** | ***median after printing [mm]*** | **deviation**  **[%]** | ***median***  ***after autoclaving process [mm]*** | **deviation**  **[%]** |
| --- | --- | --- | --- | --- | --- | --- |
| Standard |  | length  width  depth  edge- length | 50  40  10  10 | 0  0  0  0 | 50  40  10  10 | 0  0  0  0 |
| PLA 30% | 45 | length  width  depth  edge- length | 50,13  40,27  10,31  9,89 | + 0,26  + 0,65  + 3,1  - 1,1 | 48.45  38.98  10.20  9.88 | - 3,19  - 3,15  - 0,58  - 0,3 |
| PLA 100% | 45 | length  width  depth  edge- length | 50,42  40,62  10,80  9,51 | + 0,84  + 1,63  + 8,1  - 4,7 | 50.13  40.48  10.89  9.44 | - 0,52  - 0,42  + 0,83  - 0,84 |
| GreenTEC Pro®30% | 45 | length  width  depth  edge- length | 49,85  40,05  10,17  10,03 | - 0,18  + 0,2  + 1,6  + 0,1 | 49.74  39.99  10.21  9.96 | - 0,22  - 0,12  + 0,39  - 1,1 |
| GreenTEC Pro®100% | 45 | length  width  depth  edge- length | 49,98  40,08  10,08  9,96 | - 0,12  + 0,3  + 0,9  - 0,6 | 49.93  40.06  10.08  9.91 | - 0,1  - 0,1  - 0,1  - 0,6 |
